# Creatinine-Based Estimations of Kidney Function Are Unreliable in Obese Kidney Donors

**DOI:** 10.1155/2012/872894

**Published:** 2012-01-19

**Authors:** Nidhi Aggarwal, Anna C. Porter, Ignatius Y. S. Tang, Bryan N. Becker, Sanjeev K. Akkina

**Affiliations:** Department of Medicine, University of Illinois at Chicago, 820 S. Wood Street, M/C 793, Chicago, IL 60612-7315, USA

## Abstract

Accurate assessment of kidney function by measurement of glomerular filtration rate (GFR) is essential to the risk assessment of prospective living kidney donors. We evaluated the performance of various estimating equations for creatinine clearance (Cockcroft-Gault), GFR (Modification of Diet in Renal Disease, Chronic Kidney Disease Epidemiology Collaboration), and 24-hour urine collections for creatinine clearance in obese potential kidney donors. We evaluated 164 potential kidney donors including 49 with a BMI of 30–35 and 32 with a BMI >35 that have completed a routine living donor evaluation with a measured GFR. All the estimating equations performed poorly in obese donors. While 24-hour urine collections performed better, only 15% had an adequate 24-hour urine collection. Since obese kidney donors may be at higher than average risk for kidney failure, accurate assessment of kidney function in these donors is crucial to ensure their long-term health postdonation.

## 1. Introduction

Kidney transplantation is considered the treatment of choice for selected patients with end-stage kidney disease. Successful transplantation not only provides a better quality of life but also survival advantage to these patients [1]. As such, there is an increasing demand for organs but limited supply. When compared to deceased donor transplantation, living donor kidneys provide better long-term patient and graft survival rates, shorter wait times, as well as an opportunity for early or preemptive transplant [2]. In addition, transplant surgery can be performed electively and the graft usually shows prompt function postoperatively.

Prospective living donors undergo extensive medical and psychosocial evaluation to ensure that donation is safe for both recipient and donor. Most transplant programs have used a glomerular filtration rate (GFR) cut-off of 80 mL/min/1.73 m^2^ to define optimal live kidney donors [3]. Greater levels of predonation GFR are thought to leave the living donor with adequate kidney function post-donation. However, there is no consensus regarding the best method to determine GFR.

During the last two decades, there has been a dramatic rise in obesity in the United States with one-third of adults classified as obese. Not surprisingly, therefore, the proportion of donors with obesity is also rising, with 19.5% of living donors having body mass index (BMI) >30 kg/m^2^ in 2008 [4]. Most transplant programs have traditionally excluded individuals with a BMI >35 kg/m^2^ for donation due to perceived higher risk of perioperative complications [3, 5]. Increasingly, it is also recognized that obesity mediates risk in terms of kidney function, either as a direct result of excess weight or as a consequence of associated comorbidities, such as diabetes and hypertension [6–9]. Hence, obese donors should be carefully evaluated and properly educated prior to kidney donation. In this study, we evaluate the performance of estimating equations for creatinine clearance and GFR and assess the accuracy of calculated creatinine clearance by 24-hour urine collection in GFR estimation in normal to morbidly obese potential kidney donors.

## 2. Methods

### 2.1. Study Population

Individuals 18 years and older, who were evaluated as possible live kidney donors from September 1, 2009 to December 31, 2010 at the University of Illinois at Chicago were included if they had a measured glomerular filtration rate (mGFR) completed as part of their workup. The study protocol was approved by the Institutional Review Board at the University of Illinois at Chicago.

### 2.2. Estimated Glomerular Filtration Rate and Creatinine Clearance

Serum creatinine levels were measured during the initial evaluation of prospective donors. Per institutional protocol, individuals that had a BMI > 30 kg/m^2^ or an inadequate 24-hour urine collection underwent a measured GFR to assess kidney function. The estimated glomerular filtration rate (eGFR) was calculated using the 4-variable Modification of Diet in Renal Disease study equation (MDRD) [10] and the Chronic Kidney Disease Epidemiology Collaboration equation (CKD-EPI) [11].

The creatinine clearance was calculated using a 24-hour urine collection and estimated by the Cockcroft-Gault equation based on ideal body weight [12]. All results for creatinine clearance were corrected for body surface area. The 24-hour urine collection was determined to be adequate if the creatinine excretion was between 15–20 mg/kg ideal body weight for women and 20–25 mg/kg ideal body weight for men. Ideal body weight was calculated using the Devine formula [13]. Serum and urine creatinine values were measured in the clinical laboratory using the Jaffé reaction. Body surface area was calculated using the Dubois and Dubois formula [14].

### 2.3. Measured Glomerular Filtration Rate

Measured GFR (mGFR) was obtained using Technetium 99 m-mercaptoacetyltriglycine (^99 m^Tc-MAG3) renal scan. Briefly, individuals were given 7 mCi of Technetium 99 m-mercaptoacetyltriglycine while in the supine position. Dynamic images of the kidneys were obtained for 21 minutes and were read by a radiologist. Absolute GFR was reported and corrected for body surface area using the Dubois and Dubois formula [14].

### 2.4. Statistical Methods

Differences between continuous and categorical variables were compared using the one-way analysis of variance and Chi-square test, respectively. Continuous variables were reported as mean ± standard deviation. We evaluated bias that represented the average difference between measured and estimated values. A positive bias indicated that the measured value was higher than the estimated value. Precision was measured by the interquartile range (IQR) of the difference between measured and estimated values. Finally, we assessed accuracy by looking at the percentage of estimated values within 30% of measured values (P30).

## 3. Results

### 3.1. Demographics

During the study period, 164 individuals completed a ^99 m^Tc-MAG3 scan as part of their living donor evaluation. Of these individuals, 83 had a BMI < 30 kg/m^2^ (Normal group), 49 with a BMI between 30–35 kg/m^2^ (Class I Obesity group), and 32 with a BMI > 35 kg/m^2^ (Class II/III Obesity group). The characteristics of each group are shown in Table 1. Age and ethnicity distribution were similar among the three groups. There was a lower percentage of males in the Class II/III Obesity group (16%) compared to the Normal or Class I Obesity groups (51% for both).

### 3.2. Performance of GFR-Estimating Equations

Table 2 shows performance of the three GFR-estimating equations compared to mGFR in the 3 BMI groups. For the Normal group, the Cockcroft-Gault equation using ideal body weight underestimated mGFR by nearly 20 mL/min/1.73 m^2^. The MDRD and CKD-EPI eGFRs were closer to mGFR but the IQRs were similar for all three equations. The most accurate was CKD-EPI eGFR for the normal group. The performance of these equations in Class I Obesity was similar except for a lower bias for MDRD and CKD-EPI equations.

In the Class II/III Obesity group, the equations did not perform as well. The bias for MDRD and CKD-EPI eGFR suggests that the equations overestimate mGFR compared to the Normal and Class I Obesity groups. The precision was more variable between equations in this group and the accuracy was lower, especially for the MDRD and CKD-EPI equations.

### 3.3. Creatinine Clearance Measurement

All potential kidney donors also provided a 24-hour collection of urine for protein excretion and creatinine clearance. In our group of 164 individuals, 84 (51%) had overcollection, 56 (34%) had undercollection, and only 24 individuals (15%) had adequate urine collection by assessing creatinine excretion. Of these 24, the performance of the 24-hour urine collection improved for individuals with higher BMI (Table 3). In the Normal group, only 22% were within 30% of the mGFR as compared to 75% and 86% in the obese Class I and Class II/III Obesity groups, respectively.

### 3.4. Sensitivity and Specificity

Finally, we evaluated the sensitivity and specificity of the three estimating equations in selecting mGFR of 80 mL/min/1.73 m^2^ (Table 4). In the normal group, the Cockcroft-Gault equation using ideal body weight had the highest specificity while the CKD-EPI had the highest sensitivity. These findings were similar for the Class I and Class II/III Obesity groups.

## 4. Discussion

The primary goal of the extensive medical evaluation for living kidney donors is to protect the well-being of the prospective donor. Central to the donor evaluation is the assessment of predonation kidney function and factors that may affect it postdonation. Reports of long-term followup after uninephrectomy suggest that kidney donation is safe and does not adversely affect the survival or kidney function of a healthy donor [15–20]. However, efforts to expand the donor pool have led to changes in the living donor profiles from young, healthy individuals to the inclusion of older individuals with isolated medical abnormalities such as hypertension, nephrolithiasis, dyslipidemia, abnormal glucose tolerance, or obesity [3]. Data available on the long-term outcomes in such medically complex donors are still limited.

There is increasing evidence that obese individuals are at a higher risk of developing kidney disease [6–9]. Even without nephrectomy, obesity is associated with the development of hypertension and proteinuria. Higher baseline BMI is an independent predictor for development of kidney failure and this risk is directly associated with increasing levels of BMI [21]. Ibrahim et al. also showed that older donor age and higher BMI were associated with lower GFR and increased risk of hypertension, even though most donors had preserved renal function with rates of hypertension and proteinuria similar to the general population [16]. In a small study from Spain, obesity was a risk factor for the development of proteinuria and kidney failure after unilateral nephrectomy [22]. Rook et al. also demonstrated that obese individuals had lower kidney reserve capacity that was unmasked by donor nephrectomy [23]. Furthermore, Tavakol and group also found increased incidence of hypertension in obese donors though it was not associated with long-term impairments in kidney function in this study [24]. Given the limited degree of long-term followup in higher risk donors including obese individuals, it is incumbent on transplant programs that consider obese donor candidates to accurately assess kidney function of these donors to avoid future harm to these individuals.

Measured GFR using urinary or plasma clearance of exogenous filtration markers is the present gold standard for the evaluation of donor kidney function [25]. However, due to the invasive nature and complexity of these techniques, most transplant programs evaluate their kidney donors by endogenous creatinine clearance and GFR-estimating equations. The results of our study show that commonly used estimating equations for creatinine clearance and GFR perform poorly as donor BMI increases. In addition, many potential donors were unable to complete an adequate 24-hour urine collection for estimating creatinine clearance. This is consistent with the findings of other studies evaluating methods for estimating donor kidney function [26]. We also found that accurate 24-hour urine collection for creatinine clearance is difficult to obtain and thus, it is an inefficient method to assess donor GFR. Additionally, expected creatinine excretion must be calculated according to ideal body weight, not total body weight, a consideration particularly important in obese donors.

An interesting finding in our donor cohort was the negative bias seen with the MDRD and CKD-EPI equations in Class II/III obesity indicating an overestimation of GFR compared to the measured GFR. In healthy individuals, the MDRD equation is known to underestimate measured GFR [27, 28], similar to what we found in our normal BMI population. However, the overestimation in Class II/III obesity has not been reported in healthy individuals. There may be several possible explanations for this discrepancy between the measured and estimated GFR. In our cohort, women are overrepresented in the Class II/III Obesity group. Women typically have higher body fat content and less muscle mass, which would lead to lower serum creatinine values and higher eGFR. Another consideration is the applicability of estimating equations in Class II/III Obesity, especially among individuals with presumably normal kidney function. The MDRD population was mainly composed of individuals with chronic kidney disease and a mean weight of 79.6 ± 16.8 kg which was much lower than our Class I and Class II/III Obesity groups. The CKD-EPI study included more individuals with normal kidney function (kidney donors) and a higher weight and BMI of 82 ± 20 kg and 28 ± 6 kg/m^2^, respectively. While the MDRD and CKD-EPI eGFRs showed reasonable accuracy and less bias with Class I Obesity, extrapolation to Class II/III Obesity revealed a greater difference between measured and estimated GFRs and less accuracy.

One limitation of this study is the use of ^99 m^Tc-MAG3 scanning. Measured GFR using exogenous filtration markers (^125^I-iothalamate, iohexol, diethylenetriaminopenta-acetic acid) provides potentially greater accuracy of GFR assessment [25]. However, these tests may not be readily available at most centers. Moreover, additional information obtained using the ^99 m^Tc-MAG3 scanning can be helpful in identifying other aspects of kidney function and blood flow. The extents to which these parameters are either predictive or helpful in determining kidney function in the donor (or recipient) over time are not yet defined in their entirety. Another limitation is the potential selection bias among those with 24-hour urine collections. Since individuals with a normal BMI are more likely to get a measured GFR due to inadequate 24-hour urine collections, the percentage of donors with an adequate collection will be affected. However, the percentage of adequate collections in the Class I and Class II/III Obesity groups too was below 30%, indicating this to be a common problem among all three groups.

Potential concerns for transplant centers are that GFR measurement using ^99 m^Tc-MAG3 (or another type of radioisotope scan) introduces radiation exposure to the donor, cost to the evaluation process, and requires personnel familiar with the testing procedure. However, less invasive and less expensive methods may not be adequate to accurately measure kidney function in obese donors.

Although the focus of this discussion has been on the importance of accurate predonation GFR estimation for the donor, donor GFR estimation is certainly a variable of importance for the recipient as well. Lower levels of donor GFR are associated with graft dysfunction and graft loss [26, 29–31]. More accurate identification of donors with borderline kidney function prior to nephrectomy will at least inform donor and recipient decision-making regarding the surgery, as well as possibly identify recipients at increased risk for poor outcomes.

## 5. Conclusions

Accurate determination of GFR is essential to the risk assessment of prospective kidney donors. Creatinine-based GFR estimation equations are unreliable for the evaluation of obese donors. 24-hour urine collection is not only cumbersome but also fraught with errors due to inadequate collection in such donors. Further studies are required to develop better methods of GFR estimation in obese individuals with normal kidney function. Until then, measured GFR should be used as a confirmatory test to enhance clinical decision making in these circumstances.

## Figures and Tables

**Table 1:** Characteristics of potential kidney donors with measured glomerular filtration rate.

	Normal (BMI < 30) (*n* = 83)	Class I Obesity (BMI 30–35) (*n* = 49)	Class II/III Obesity (BMI > 35) (*n* = 32)	*P* value
Age (yrs)	39.1 ± 12.4	39.1 ± 10.0	35.7 ± 9.7	0.30
Weight (kg)	76.5 ± 10.7	96.1 ± 10.9	107.6 ± 16.3	<0.001
BMI	26.5 ± 2.5	32.3 ± 1.4	40.0 ± 4.3	<0.001
Male	42 (51%)	25 (51%)	5 (16%)	0.002
Ethnicity				0.14
White	24 (29%)	9 (18%)	5 (16%)	
Black	30 (36%)	21 (43%)	20 (62%)	
Hispanic	24 (29%)	13 (27%)	6 (19%)	
Other	5 (6%)	6 (12%)	1 (3%)	

Abbreviations: BMI—body mass index (kg/m^2^).

**Table 2:** Performance of estimating equations.

	Bias (mL/min/1.73 m^2^)	IQR (mL/min/1.73 m^2^)	P30
Normal (BMI < 30) (*n* = 83)			
Cockcroft-Gault CrCl	19.7 ± 2.6	35.3	64%
MDRD eGFR	10.2 ± 2.7	35.6	71%
CKD-EPI eGFR	6.5 ± 2.7	35.3	76%

Class I Obesity (BMI 30–35) (*n* = 49)			
Cockcroft-Gault CrCl	17.4 ± 3.6	36.5	61%
MDRD eGFR	0.54 ± 3.9	36.0	69%
CKD-EPI eGFR	−3.0 ± 3.9	37.9	69%

Class II/III Obesity (BMI > 35) (*n* = 32)			
Cockcroft-Gault CrCl	15.9 ± 3.8	31.9	66%
MDRD eGFR	−15.0 ± 4.3	37.3	62%
CKD-EPI eGFR	−19.2 ± 4.1	35.7	56%

Abbreviations: Bias—(measured GF-estimated GFR); IQR—interquartile range; BMI—body mass index (kg/m^2^); P30—percentage of estimated readings within 30 percent of measured reading; CrCl—creatinine clearance; MDRD—modification of diet in renal disease; CKD-EPI—chronic kidney disease epidemiology collaboration; eGFR—estimated glomerular filtration rate.

**Table 3:** Performance of 24-hour creatinine clearance.

	Bias (mL/min/1.73 m^2^)	IQR (mL/min/1.73 m^2^)	P30
Normal (BMI < 30) (*n* = 9)	23.6 ± 9.2	38.8	22%
Class I Obesity (BMI 30–35) (*n* = 8)	17.8 ± 6.5	23.9	75%
Class II/III Obesity (BMI > 35) (*n* = 7)	6.1 ± 4.2	10.0	86%

Abbreviations: Bias—(measured GF-estimated GFR); IQR—interquartile range; BMI—body mass index (kg/m^2^); P30—percentage of estimated readings within 30 percent of measured readings.

**Table 4:** Sensitivity and specificity of estimating equations.

	mGFR > 80 mL/min/1.73 m^2^	
	SENS	SPEC
Normal		
CG-CrCl	51%	67%
MDRD eGFR	74%	47%
CKD-EPI eGFR	81%	40%

Class I Obesity		
CG-CrCl	47%	69%
MDRD eGFR	81%	46%
CKD-EPI eGFR	86%	23%

Class II/III Obesity		
CG-CrCl	45%	92%
MDRD eGFR	100%	17%
CKD-EPI eGFR	100%	17%

Abbreviations: mGFR—measured glomerular filtration rate; SENS—sensitivity; SPEC—specificity; CG-Cockcroft-Gault equation; CrCl—creatinine clearance; MDRD—modification of diet in renal disease; CKD-EPI—chronic kidney disease epidemiology collaboration; eGFR—estimated glomerular filtration rate.
